# Association between hypertension and coffee drinking based on CYP1A2 rs762551 single nucleotide polymorphism in Taiwanese

**DOI:** 10.1186/s12986-021-00605-9

**Published:** 2021-08-14

**Authors:** Chien-Chou Hou, Disline Manli Tantoh, Chuan-Chao Lin, Pei-Hsin Chen, Hao-Jan Yang, Yung-Po Liaw

**Affiliations:** 1 Department of Medical Sociology and Social Work, Chung Shan Medical University, Taichung City, Taiwan; 2 Department of Social Service, Chung Shan Medical University Hospital, Taichung City, Taiwan; 3 Department of Medical Imaging, Chung Shan Medical University Hospital, Taichung City, Taiwan; 4 Department of Public Health and Institute of Public Health, Chung Shan Medical University, No. 110 Sec. 1 Jianguo N. Road, Taichung City, 40201 Taiwan; 5 Department of Physical Medicine and Rehabilitation, Chung Shan Medical University Hospital, Taichung City, Taiwan; 6 School of Medicine, Chung Shan Medical University, Taichung City, Taiwan; 7 Department of Family and Community Medicine, Chung Shan Medical University Hospital, Taichung City, Taiwan

**Keywords:** Hypertension, Coffee drinking, CYP1A2, rs762551, Taiwanese

## Abstract

**Background:**

Hypertension increases the likelihood of cardiovascular diseases (CVDs). Cytochrome P450 1A2 (CYP1A2) single nucleotide polymorphism (SNP) is related to caffeine metabolism and the risk of CVD among coffee drinkers. CYP1A2 rs762551 influenced the risk of stroke among hypertensive patients. We examined the relationship between hypertension and coffee drinking based on CYP1A2 rs762551 SNP in Taiwanese adults.

**Methods:**

We used data contained in the Taiwan Biobank database (2011–2018) and included 19,133 participants having complete information on hypertension, rs762551 polymorphism, coffee intake, etc. The risk of hypertension was determined using multiple logistic regression.

**Results:**

Coffee intake was significantly associated with a lower risk of hypertension. The odds ratio (OR), 95% confidence interval (CI), and p-value were 0.877, 0.807–0.954, and 0.0032, respectively. CYP1A2 rs762551 was not significantly associated with the risk of hypertension, but it had a significant interactive association with coffee drinking (*p* value = 0.0303). After stratification by rs762551 genotypes, the inverse coffee drinking-hypertension association was retained, but significant results were observed only in those with the AC + CC genotype (OR 0.678, 95% CI 0.722–900, *p* value = 0.0001). According to the combination of coffee drinking and rs762551 genotypes (reference group: no coffee drinking and rs762551 AA), the coffee drinking-AC + CC group had a lower risk of hypertension (OR 0.888, 95% CI 0.789–0.999, *p* value = 0.0483).

**Conclusion:**

Coffee drinking, particularly among individuals with the CYP1A2 rs762551 AC + CC genotype was associated with lower odds of hypertension.

## Background

Hypertension is a significant public health burden. Its prevalence among adults has been increasing. In 2000, about 972 million adults in the world were believed to be hypertensive [1]. However, by 2010, hypertension among adults was estimated at 1.4 billion [2]. It is projected at about 1.56 billion by 2025 [1]. The disease is multiplying rapidly in Asia, and the prevalence rate in Taiwan is about 25% in men and 18% in women [3]. Hypertension increases the likelihood of CVDs like heart failure, stroke, and coronary heart disease [1, 4–6], which account for a considerable number of global deaths [7, 8]. The burden of deaths attributable to CVDs was approximately 12.3 and 17.3 million in 1990 and 2013, respectively [7], and 17.92 million in 2015 [8]. Some cardiovascular outcomes are believed to be the consequence of the effect of coffee on blood pressure (BP) [9].

Coffee consumption has been increasing in Taiwan and the world [10, 11]. Dietary and lifestyle factors play substantial roles in modifying the risk of hypertension [12]. A previous study indicated that regular consumption of three or more cups of coffee a day was protective against hypertension [13]. In the SUN project, regular consumption of coffee was associated with a decreased risk of hypertension (about 26%) only in women [14]. A meta-analysis suggested that long-term consumption of coffee may lower the incidence of hypertension [15]. Daily intake of green or black tea was associated with a modest increase in hypertension risk [16]. However, the risk became non-significant when caffeine was adjusted for in the model. Marjan and colleagues found that regular intake of tea reduced systolic blood pressure (SBP) and diastolic blood pressure (DBP) by 4.81 mmHg and 1.98 mmHg, respectively [17].

So far, findings from epidemiological research on coffee drinking and hypertension have been controversial [4]. Some short-term intervention studies revealed a positive association [18]. However, one prospective study found that coffee intake was beneficial for hypertension only among non-smokers [19] while several prospective studies found that coffee intake could elevate the risk of hypertension [20–23].

Genetic factors contribute to variability in the effect of coffee on cardiovascular health [24]. Specific genetic polymorphisms related to caffeine metabolism pose a risk of CVD among coffee drinkers. For instance, polymorphism at CYP1A2 rs762551 was associated with impaired caffeine metabolism, owing to low enzyme actions [25]. This variant was also associated with stroke risk in a Chinese population with hypertension, cerebral infarction, and coronary heart disease [26]. About 95% of caffeine metabolism is attributed to CYP1A2 [27]. CYP1A2 modulated the effect of caffeinated coffee on myocardial infarction [28] and its polymorphism was associated with hypertension [29]. Moreover, caffeine intake modified the association between CYP1A2 variants and blood pressure (BP) among European adults [30, 31]. Research on hypertension based on CYP1A2 polymorphism and coffee intake in Taiwanese is seemingly rare. Considering the controversial influence of coffee on hypertension and cardiovascular health, and the importance of genetic insights in the management of diseases in this era, we undertook this study to evaluate the relationship between hypertension and coffee drinking based on CYP1A2 rs762551 single nucleotide polymorphism in Taiwanese.

## Methods

### Data source

We conducted this study using data contained in the Taiwan Biobank database (2011–2018). The biobank contains genetic data as well as information like medical history, sex, age, diet, and other lifestyle factors. Enrolment into the Taiwan Biobank project is restricted to Taiwanese adults between 30 and 70 years without a personal history of cancer.

### Study population and variable definitions

Variables included hypertension (yes/no), coffee drinking (yes/no), rs762551 genotypes (AA/AC + CC), tea drinking (yes/no), sex (men/women), age, body mass index or simply BMI (underweight/normal /overweight/obese), cigarette smoking (never/former/current), alcohol drinking (never/former/current), and exercise (no/yes). Hypertensive cases were those who confirmed through questionnaires to have ever been clinically diagnosed with the disease. Coffee drinking was defined according to self-reports on weekly consumption frequency. The coffee-drinking group included individuals who self-reported a habit of drinking coffee at least thrice per week while the non-drinking group included those who habitually drank coffee less than three times per week. Tea drinking was defined based on daily consumption frequency. The tea-drinking category included those who self-reported a habitual daily tea intake of at least one time while the non-drinking category included those who had a habit of not drinking tea every day. Sex and age were self-reported while BMI was derived from weight (kg) and height (m) using the standard formula: weight divided by (height)^2^. Underweight was defined as BMI < 18.5 kg/m^2^, normal BMI as 18.5 ≤ BMI < 24 kg/m^2^, overweight as 24 ≤ BMI < 27 kg/m^2^, and obesity as BMI ≥ 27 kg/m^2^. Participants were considered non-smokers if they had no history of cigarette smoking for continuously 6 months or more. Former smokers were those who smoked cigarettes continuously for at least 6 months but were not smoking during data collection. Current smokers were those who had a history of continuous cigarette smoking for at least 6 months and were still smoking during data collection. Participants were considered non-drinkers if they had no history of continuous drinking for 6 months or drank less than 150 cc of alcohol per week. Former drinkers were those who abstained from alcohol for more than 6 months while current drinkers were those who drank at least 150 cc of alcohol per week continuously for 6 months. Exercise was defined as regular exercise taken 3 times a week (with each session lasting over 30 min). This study included 3411 hypertensive and 15,722 non-hypertensive individuals. All participants signed an informed consent letter. We obtained ethical approval from the Institutional Review Board of Chung Shan Medical University (CS1-20,009).

### Statistical analyses

CYP1A2 rs762551 was validated using quality control measures including, Hardy–Weinberg equilibrium *p* > 0.001, a minor allele frequency ≥ 0.05, and call rate ≥ 0.05. Quality control was performed using PLINK version 1.9. Chi-square test was used to differentiate the categorical variables while Student’s *T* test was used to differentiate the continuous variables between cases and non-cases. Categorical variables were presented as n (percentage) and continuous variables as mean ± standard error. Multivariate logistic regression was used to evaluate the association of coffee drinking and rs762551 with hypertension and also the interaction between coffee drinking and rs762551. Statistical analyses were performed using SAS version 9.4 (SAS Institute Inc., Cary, North Carolina, USA), and *p* values less than 0.05 were considered to be statistically significant.

## Results

Table 1 shows the demographic data of 19,133 participants stratified as coffee drinkers (n = 7766) and non-drinkers (n = 11,367). Of the 19,133 participants, 3411 were hypertensive while 15,722 were non-hypertensive. Coffee drinkers and non-drinkers significantly differed according to hypertension (*p* value < 0.0001) but not according to rs762551 genotypes (*p* value = 0.8659). Coffee drinkers and non-drinkers also differed in terms of tea drinking (*p* value < 0.0001), sex (*p* value = 0.0154), age, BMI, cigarette smoking, and alcohol drinking (*p* value < 0.0001) but not exercise (*p* value = 0.2581).

**Table 1:** Demographic data of participants according to coffee drinking habits

Variables	No coffee drinking (n = 11,367)	Coffee drinking (n = 7766)	*p* value
*Hypertension*			< 0.0001
No	9215(81.07)	6507(83.79)	
Yes	2152(18.93)	1259(16.21)	
*rs762551 genotype*			0.8659
AA	4844(42.61)	3319(42.74)	
AC + CC	6523(57.39)	4447(57.26)	
*Tea drinking*			< 0.0001
No	8859(77.94)	5610(72.24)	
Yes	2508(22.06)	2156(27.76)	
*Sex*			0.0154
Women	7277(64.02)	5104(65.72)	
Men	4090(35.98)	2662(34.28)	
Age (years)	55.70 ± 0.0951	54.08 ± 0.1125	< 0.0001
*BMI*			< 0.0001
Underweight	364(3.20)	169(2.18)	
Normal	5544(48.77)	3653(47.04)	
Overweight	3286(28.91)	2332(30.03)	
Obesity	2173(19.12)	1612(20.76)	
*Cigarette smoking*			< 0.0001
Never	9385(82.56)	6145(79.13)	
Former	1212(10.66)	997(12.84)	
Current	770(6.77)	624(8.04)	
*Alcohol drinking*			< 0.0001
Never	10,234(90.03)	6825(87.88)	
Former	461(4.06)	309(3.98)	
Current	672(5.91)	632(8.14)	
*Exercise*			0.2581
No	5766(50.73)	4004(51.56)	
Yes	5601(49.27)	3762(48.44)	

Table 2 shows the association of hypertension with coffee and other variables. Coffee intake was significantly associated with a lower (OR 0.877, 95% CI 0.807–0.954, *p* value = 0.0032) while the rs762551 variant was associated with a higher but not significant risk of hypertension (OR 1.021, 95% CI 0.941–1.107, *p* value = 0.6176).

**Table 2:** Multiple logistic regression model showing the association between coffee drinking and hypertension

Variables	OR	95% CI	p-value
*Coffee drinking (Reference: No)*			
Yes	0.877	0.807–0.954	0.0032
*rs762551 genotype (Reference: AA)*			
AC + CC	1.021	0.941–1.107	0.6176
*Tea drinking (Reference: No)*			
Yes	0.988	0.899–1.086	0.8002
*Sex (Reference: Women)*			
Men	1.241	1.123–1.371	< 0.0001
Age	1.094	1.088–1.099	< 0.0001
*BMI (Reference: Normal)*			
Underweight	0.222	0.129–0.382	< 0.0001
Overweight	2.018	1.831–2.224	< 0.0001
Obesity	4.421	3.989–4.901	< 0.0001
*Cigarette smoking (Reference: Never)*			
Former	1.159	1.016–1.323	0.0283
Current	1.010	0.849–1.201	0.9119
*Alcohol drinking (Reference: Never)*			
Former	1.502	1.253–1.800	< 0.0001
Current	1.366	1.166–1.601	0.0001
*Exercise(Reference: No)*			
Yes	1.032	0.947–1.123	0.4737

OR: odds ratio, CI: confidence interval

The rs762551 variant had a significant interaction with coffee (*p* value = 0.0303) but not with tea (*p* value = 0.4183) as seen in Table 3. After stratification by rs762551 genotypes, coffee drinking was significantly associated with a lower risk of hypertension only in those with the AC + CC genotype (OR 0.806, 95% CI 0.722–0.900, *p* value  = 0.0001). Further stratification of coffee drinking revealed a significant dose–response association (*p* value < 0.0001) between coffee drinking and hypertension in those carrying the AC + CC genotype (Table 4).

**Table 3:** Multiple logistic regression model showing the association between coffee drinking and hypertension grouped into the rs762551 genotypes

Variables	rs762551 AA			rs762551 AC + CC		
	OR	95% CI	p-value	OR	95% CI	p-value
*Coffee drinking (Reference: No)*						
Yes	0.979	0.862–1.112	0.7433	0.806	0.722–0.900	0.0001
*Tea drinking (Reference: No)*						
Yes	0.945	0.817–1.092	0.4419	1.024	0.905–1.160	0.7034
*Sex (Reference: Women)*						
Men	1.256	1.078–1.464	0.0035	1.229	1.078–1.402	0.0021
Age	1.096	1.088–1.105	< 0.0001	1.092	1.084–1.099	< 0.0001
*BMI (Reference: Normal)*						
Underweight	0.285	0.131–0.616	0.0014	0.183	0.086–0.392	< 0.0001
Overweight	1.910	1.646–2.216	< 0.0001	2.100	1.847–2.389	< 0.0001
Obesity	4.290	3.659–5.030	< 0.0001	4.534	3.960–5.190	< 0.0001
*Cigarette smoking (Reference: Never)*						
Former	1.200	0.980–1.469	0.0779	1.133	0.951–1.349	0.1612
Current	0.882	0.675–1.153	0.3596	1.121	0.894–1.407	0.3225
*Alcohol drinking (Reference: Never)*						
Former	1.515	1.142–2.009	0.0039	1.482	1.170–1.878	0.0011
Current	1.471	1.154–1.876	0.0018	1.283	1.040–1.584	0.0199
*Exercise(Reference: No)*						
Yes	1.006	0.883–1.145	0.9341	1.051	0.939–1.177	0.3839

Interactions coffee drinking*rs762551 p-value = 0.0303, tea drinking*rs762551 p-value = 0.4183. OR: odds ratio, CI: confidence interval

**Table 4:** Multiple logistic regression model showing the dose–response association between coffee drinking and hypertension grouped into the rs762551 genotypes

Variables	rs762551 AA			rs762551 AC + CC		
	OR	95% CI	p-value	OR	95% CI	p-value
*Coffee drinking* *(Reference: no coffee drinking)*						
< 1800 ml/week	1.029	0.879–1.205	0.7232	0.856	0.745–0.983	0.0278
≥ 1800 ml/week	0.916	0.776–1.081	0.2977	0.755	0.654–0.872	0.0001
Trend test			0.4037			< 0.0001
*Tea drinking (Reference: No)*						
Yes	0.937	0.810–1.084	0.3817	1.025	0.905–1.161	0.6934
*Sex (Reference: Women)*						
Men	1.256	1.078–1.465	0.0035	1.224	1.073–1.396	0.0026
Age	1.096	1.087–1.105	< 0.0001	1.091	1.084–1.099	< 0.0001
*BMI (Reference: Normal)*						
Underweight	0.285	0.131–0.616	0.0014	0.182	0.085–0.390	< 0.0001
Overweight	1.906	1.642–2.212	< 0.0001	2.101	1.847–2.390	< 0.0001
Obesity	4.317	3.681–5.062	< 0.0001	4.537	3.963–5.195	< 0.0001
*Cigarette smoking (Reference: Never)*						
Former	1.191	0.972–1.459	0.0921	1.137	0.955–1.354	0.1488
Current	0.880	0.672–1.151	0.3512	1.126	0.897–1.415	0.3059
*Alcohol drinking (Reference: Never)*						
Former	1.528	1.153–2.027	0.0032	1.492	1.177–1.891	0.0009
Current	1.480	1.160–1.888	0.0016	1.284	1.041–1.585	0.0197
*Exercise(Reference: No)*						
Yes	1.008	0.885–1.148	0.9073	1.051	0.939–1.177	0.3854

OR: odds ratio, CI: confidence interval

According to the combination of coffee drinking and the rs762551 genotypes (Table 5) with the reference group being no coffee drinking and the rs762551 AA genotype, the risk of hypertension was lower in the coffee drinking and AC + CC group (OR 0.888, CI 0.789–0.999, *p* value = 0.0483).

**Table 5:** Multiple logistic regression model showing the odds ratios for hypertension according to the combination of coffee drinking and the rs762551 genotypes

Variables	OR	95% CI	*p* value
*Coffee drinking, rs762551 genotype (Reference: No coffee drinking and AA)*			
No coffee drinking and AC + CC	1.095	0.988–1.215	0.0844
Coffee drinking and AA	0.975	0.859–1.107	0.6960
Coffee drinking and AC + CC	0.888	0.789–0.999	0.0483
*Tea drinking (Reference: No)*			
Yes	0.989	0.900–1.087	0.8222
*Sex (Reference: Women)*			
Men	1.239	1.122–1.369	< 0.0001
Age	1.094	1.088–1.099	< 0.0001
*BMI (Reference: Normal)*			
Underweight	0.222	0.129–0.382	< 0.0001
Overweight	2.017	1.830–2.223	< 0.0001
Obesity	4.430	3.997–4.911	< 0.0001
*Cigarette smoking (Reference: Never)*			
Former	1.161	1.017–1.325	0.0266
Current	1.013	0.852–1.204	0.8850
*Alcohol drinking (Reference: Never)*			
Former	1.499	1.251–1.797	< 0.0001
Current	1.364	1.164–1.599	0.0001
*Exercise(Reference: No)*			
Yes	1.032	0.948–1.124	0.4693

OR: odds ratio, CI: confidence interval

## Discussion

We used the 2011–2018 Taiwan Biobank cohort in the current study. Descriptive data showed that coffee drinkers and non-drinkers significantly differed according to hypertension but not according to the rs762551 genotypes. Logistic regression analysis also revealed a non-significant association between AC + CC genotype (reference: AA) and coffee drinking (data not shown). After multivariate logistic regression analysis, coffee drinking had a significant inverse association with hypertension. CYP1A2 rs762551 was not significantly associated with the risk of hypertension but had a significant interaction with coffee drinking. After stratifying participants by rs762551 genotypes, the inverse coffee-hypertension association was retained only among those with the AC + CC genotype. Combining coffee and rs762551 genotypes revealed a lower risk of hypertension among coffee drinkers with the AC + CC genotype. The inverse association between coffee drinking and hypertension in the AC + CC group was in a dose-dependent manner.

The non-significant association between coffee drinking and rs762551 genotype observed in the current study is in concordance with previous reports among Costa Ricans [28] and Italians [29]. Contrarily, coffee drinking was significantly lower among Japanese with the AC + CC genotype [32]. In a meta-analysis, coffee drinking among Asians was not significantly associated with the rs762551 genotypes [33]. On the other hand, the aforementioned meta-analysis revealed that coffee drinking was significantly higher among Caucasians carrying the rs762551 AA genotype compared to the AC + CC genotype [33].

The relationship between coffee and hypertension has been controversial. For instance, a meta-analysis of randomized clinical trials demonstrated a positive but weak association between coffee intake and hypertension [21]. In a follow-up study, coffee intake was associated with higher blood pressure but played a very minute role in the onset of hypertension [34]. In another follow-up study, there was no significant linear association between coffee drinking and hypertension [35]. While a lower risk of hypertension was observed in men who abstained from coffee compared to those who drank moderate amounts, heavy coffee consumption among women was associated with a lower risk of hypertension [36]. In conformity to our observation, results from a Mendelian randomization analysis revealed an inverse association between coffee drinking and hypertension [30]. The seemingly protective effect of coffee against hypertension could in part elucidate why coffee drinking is associated with a lower risk of stroke [37, 38]. Coffee drinking is also associated with other cardiovascular health benefits, including protection against cerebral infarction [39], coronary heart disease [40], and CVD-related/all-cause mortality [10]. These beneficial effects of coffee are attributed to its richness in polyphenols which possess antihypertensive, antioxidant, and anti-inflammatory properties [16, 41].

The observed differences in the relationship between coffee drinking and hypertension are in part due to genetics [42]. Coffee consumption was associated with an increase in CYP1A2 enzyme activity [43]. Moreover, the association of hypertension with coffee drinking was also confirmed to vary according to CYP1A2 genotypes [29]. AA homozygotes compared to AC individuals have greater enzyme activities and are believed to metabolize caffeine relatively fast [25, 44]. Both moderate and heavy coffee consumption among Italians carrying the C allele appeared to confer higher susceptibility to hypertension [29]. Swedish individuals with higher caffeine intake carrying the A allele were more likely to have a lower BP and lower risk of hypertension than those carrying the C allele [30]. In the present study, coffee drinking among Taiwanese adults aged 30–70 years carrying the AC + CC genotypes was associated with a lower risk of hypertension. The discrepancy in findings could be due to differences in sample sizes, age, and ethnicity. In the study of Palatino and colleagues (2009), only 553 individuals were included in the study. Moreover, the age range of the participants was 18–45 years [29]. We had a relatively large sample size (n = 19,133) and the age-range was 30–70 years. It was proposed that the CYP1A2 enzyme affects BP by differentially inhibiting adenosine receptors (42).

Despite the large sample size, our study is limited in that the TWB questionnaires contained no information on caffeine intake and coffee type. Therefore, we could not determine the interaction between CYP1A2 polymorphism and caffeine or decaffeinated coffee on hypertension risk. Moreover, we defined hypertension based on the Taiwan Biobank questionnaires (as described in the Methods section). This might have resulted in information bias. However, it should be noted that in our initial analyses, we merged the Taiwan Biobank database with the National Health Insurance Research Database (NHIRD) using encrypted personal identification numbers with the resources at the Health and Welfare Data Science Center (HWDC). The NHIRD contained diagnostic codes based on the International Classification of Diseases, Ninth Revision, Clinical Modification (ICD-9-CM). Hypertension was identified based on either two outpatient visits or one-time hospitalization between 1998 and 2015 using the diagnostic codes ICD-9-CM 401-405, A260, and A269. Even though we defined hypertension based on self-reports, all cases were those whose diagnosis was confirmed by a medical doctor. Noteworthy, the results observed are similar to those observed when hypertension was defined using ICD codes.

## Conclusion

Generally, coffee drinking was associated with a lower risk of hypertension, while the AC + CC genotype was associated with a higher but non-significant risk. Notwithstanding, coffee drinking and rs762551 had a significant interactive relationship. Grouped analyses revealed an inverse association between hypertension and coffee drinking, particularly among individuals with the CYP1A2 rs762551 AC + CC genotype. Our results indicate the possibility of a protective effect of coffee on hypertension and confirm the joint role of coffee and CYP1A2 rs762551 polymorphism on hypertension.

## Abbreviations

BMI: Body mass index
OR: Odds ratio
CVD: Cardiovascular disease
CYP1A2: Cytochrome P450 1A2
SNP: Single nucleotide polymorphism
NHIRD: National Health Insurance Research Database
ICD-9-CM: International Classification of Diseases, Ninth Revision, Clinical Modification
HWDC: Health and Welfare Data Science Center

## Declarations

### Availability of data and materials

The data that support the findings of this study are available from Taiwan Biobank but restrictions apply to the availability of these data, which were used under license for the current study, and so are not publicly available. Data are however available from Professor Yung Po Liaw (Email address: Liawyp@csmu.edu.tw, Tel: + 886424730022 ext. 12102; fax: + 886423248179) upon reasonable request and with permission of Taiwan Biobank.
